# Extracts and Varieties

**Published:** 1848-04-01

**Authors:** 


### Extracts and Varieties.

PARALYSIS OF THE TONGUE INDUCED BY PASSION.—Dr. Pupke relates the following case in the "Medicinische Zeitung:"—M. N., aged 59 years, of delicate constitution and choleric temperament, while engaged in a violent dispute with his partner, suddenly lost the power of speech, the intellectual faculties, however, remaining unimpaired. He was from this time unable to move the tongue freely, or to utter any articulate sounds. Respiration, the heart's action, and the fæcal and urinary evacuations were normal. His face was pale, and nothing particular was expressed by the features. The tongue was clean, pulse regular, but somewhat sluggish and labouring. No symptoms of congestion of the brain were observed. Dr. Pupke being called in soon after the attack, immediately had recourse to electricity, by means of magnetico-electrical rotation, two metallic chains from which conducted the agent into the paralysed organ. The electricity was employed three times a day, each operation lasting from five to ten minutes. During the operation the patient was requested to keep in his mouth a mixture of the decoctum radicis calami and tinctura radicis pyrethri. In the course of five days the organ was restored to its healthy state.

REMARKABLE POST-MORTEM EXAMINATION OF AN ELDERLY IDIOTIC FEMALE.—*Channel discovered descending between the pyramids of the spinal cord from the fourth ventricle.* By Dr. WALLIS. The patient had been an inmate of an asylum for many years, and been gradually declining up to the period of her death. The substance of the brain was atrophied, and the membranes lying over it in folds, opaque in a great part of their extent, and contained a large quantity of an aqueous liquid. The structure of the cerebrum and cerebellum was normal, but all the ventricles were extremely distended with serum. On separating the medulla oblongata from the brain, it was found that towards the centre of the two cut surfaces, where the fibres of the medulla are mutually crossing, a small opening was discovered leading to a channel, which on being traced upwards, was found to communicate at the calamus scriptorius with the fourth ventricle. This channel could also be traced descending between the pyramids of the spinal cord, even so far as this could be examined without opening the spinal canal, which Dr. Wallis was not permitted to do. The author states that he has on three former occasions met with similar channels in cases of hydropsy of the cavities of the brain, but this is the first instance, so far as he is aware, in which such a condition was found to exist in an adult. Dr. Wallis remarks that he subsequently became aware that Stilling and Wallach had demonstrated the presence of a channel in the spinal cord of children, which he had previously observed, which fully establishes the existence of a channel in the spinal marrow, communicating with the ventricles of the brain, which becomes, as in this case, visible in dropsy of the brain and spinal cord.

Plea of insanity.—Mania is one of the most frequent subjects of forensic inquiry in which the physician is called on to decide; and, to the disgrace of science, we find the most opposite opinions adduced by practitioners of eminence. Much depends on the period during which the physician sees the supposed lunatic, and more on a few necessary distinctions, which, we fear, are sometimes designedly neglected. It is possible for an interested relation to fix on a day when the patient is calm and rational, an hour when he is usually collected, to introduce the physician who pronounces him *sane*. Another, in different circumstances, might pronounce him mad. It is necessary, therefore, to guard against such deceptions, to visit him frequently, at different times, and at the most unexpected hours. If this is refused, a collusion will be evident. We remember seeing a man who was confined for a crime, and defended on the plea of idiotic insanity. We visited him frequently, while unsuspecting any such examination, and found the plea strictly true; yet when called into court for the purpose of acquittal,—when cleaned and dressed,—roused also, perhaps, by the novel appearance of the scene,—his look assumed a meaning, and he was almost rational.

In the general relations of life, a man may be thoughtless, ridiculous, and extravagant, yet these errors will not be sufficient to fix the charge of insanity; which consists, either in false perceptions, or erroneous reasoning, on subjects distinguished in their true colours. Many individuals of this kind require guardians for their property, as much as persons really insane, but the law entrusts no practitioner with such discretionary power. The difficulty arises when this wild, absurd conduct is attended with such inconsistencies as lead to the suspicion that the perceptions or the reason are affected. This situation is a question of prudence, rather than of jurisprudence or medicine. The reflecting physician will not fix unnecessarily the stigma of insanity on a whole race, nor will he expose a family to ruin by a too great delicacy. In this difficulty, he will rather take the opportunity of a calmer moment to induce the patient to adopt such plans as may prevent the ruin of the family, and may properly make use of the alternative as an argument in case of refusal. But this, as we have said, is not a medical question.

There is another doubtful state, in which the physician is often called on to decide,—viz., when, from disease, from general weakness, or any constitutional cause, the mind is so much enfeebled as to render it uncertain whether the patient can judge of the proper disposition of his affairs. This, too, is a question of discretion; for the afflicted person may be taught to answer common questions readily, or may be awed by some interested attendant. In this case, if the physician, when alone with his patient, talks to him of his affairs, suggests—for the sake of a reply only—some objections to his arrangements, he will soon find whether the testator has judged properly, or only repeats a lesson. The circumstances themselves often suggest doubts; and when an infirm old man disinherits obedient or near relations for the sake of those connected with him only by accident, the presumption is, that his mind is not sound.

We have said, in the article, "Mania," that by a fiction of the law, every mania, we have been informed, is supposed to be relieved by occasional lucid intervals, and that, if the act of a madman is reasonable and proper, it is a proof that the interval was a lucid one. Thus, in the case which has just been considered, whatever be the state of the patient's mind, if his will be judicious and proper, there is no reason why the physician should not pronounce him in a sound state. Yet, in criminal cases, the law is not equally indulgent, nor has it always, perhaps, been equally humane. Lucid intervals, in cases of murder, are not allowed, and a man who has been proved to be mad on the Monday and Wednesday, is not allowed to be sane on the intervening day; yet decisions have occurred of a different kind, and an art in planning, a coolness in executing, a deliberation in the conduct, have been supposed to constitute soundness of mind. On these grounds, Lord Ferrers and Mr. Oliver were executed. Yet, if the motive is at any time connected with the hallucinations, the subsequent action should certainly be considered as a part. In later trials, the opinions have leant more on the side of humanity.

The question of confirmed insanity must be decided by a comparison of the patient's state with the pathognomonic symptoms. Yet there are many sources of doubt, and often room for hesitation. In many instances the mind wanders,—at first, on one subject only; and when the madman has any point to gain, he will, with great success, counterfeit a calm, reasonable state. Each point must be carefully guarded; yet the experienced physician will not be easily baffled. A wildness of the eye,—a tension of the skin of the temples,—a dry, furred tongue,—often a hurried pulse, will explain the real state. The madman is also a coward, and we have drawn from this a good pathognomonic symptom. If threatened with some vehemence with any punishment, however wild and impracticable, he will shrink and tremble, forgetting all his art, or returning to his original deviation of mind.

Returning sanity is another point of doubtful distinction, nor do we see that it is possible to lay down any rules, except the absence of the pathognomonics of the disease. Yet we have often witnessed the return of persons from the appropriate receptacles, with a wildness of the eyes, a quickness of utterance, rapid, unsteady motions, which showed corporeal disease, though the mind was calm. Such persons should not be pronounced secure, and though confinement may not be necessary, the most pointed caution should be continued.

Dissembled insanity might more properly belong to another head, *morbi simulati*, but we may more easily speak of it in this place. An experienced practitioner will soon detect the absurdities which assume the form of insanity; for though incoherencies, wildness, and obscenity may be imitated, the hurried look, the rapid pulse, the dry tongue, and the sleepless nights cannot be assumed. Above all, the cowardice, the apprehension of punishment, the influence of threats, are seldom to be discovered. A French author details the symptoms of madness, for the purpose of this distinction, so elegantly, as to induce us to copy the picture:—"Thus to neglect what most deserves attention, and to value what is least deserving of it; to rejoice or weep without an adequate reason; to despise what is terrible, and to fear what is ridiculous; to admire trifles, and to reject what is excellent; to love the objects of hate, and to hate those of love; to hope without an object, and to despair while in security; to be pleased with things which excite no agreeable sensations in others, and to fly from what every one would anxiously seek; to be timid with those who demand no deference, and bold to those whom they ought to respect; such are the infallible marks of a wandering mind."—*Art.*, "*Medicinæ Forensis et Politica*," *Parr's Lond. Med. Dict.*

BIOGRAPHICAL NOTICE OF FODÉRÉ.—(From Dacros's "Notice Historique sur la Vie et les Travaux du Dr. Fodéré.")—François Emanuel Fodéré was born January 8, 1764, at St. Jean de Maurienne, in Savoy, of humble parents. His father died at the time he was born; but his mother carefully brought him up, so as to cultivate the talents he displayed at an early age, until the intendant of St. Maurienne, Ritter Von Poniel Real, took him under his protection. During his medical studies, which he prosecuted at Turin, he directed his attention to cretinism, and the result of his labours held for a long time the first place in this branch of pathology. He graduated in 1787; and was afterwards supplied with money by Victor Amadeus III. to enable him to finish his education in the hospitals of Paris and London. On his return, he was appointed forensic physician to the Duchy of Costa. The war with the French republic breaking out, and Savoy being united to it, led to the attachment of Fodéré to the army of Italy. This did not, however, interrupt his scientific researches, inasmuch as he published an account of the diseases of the troops in the territory of Mantua. The campaign being ended, he accompanied the division of the army to which he was attached to Marseilles, and there married the daughter of Dr. Moulart, a distinguished practitioner. This happened in February, 1793; and about the same time, two of his wife's cousins were married also—the one to Joseph Bonaparte, the other to General Bernadotte. This high connexion was never of any benefit to him. After marriage, Fodéré was attached to the army of the Alps; and on his return, was appointed physician to the hospital and lunatic asylum at Marseilles, where he commenced a course of lectures on anatomy and physiology. In 1796, he published his celebrated work on forensic medicine and public hygiene. Fodéré was next professor of natural philosophy and chemistry at the central school of Nizza. In 1803, his statistics of the Alps appeared; and in the following year, he was physician to the Hôtel Dieu and asylum at Marseilles. In 1814, he was consulting physician to the captive royal family of Spain. Although past middle age, he contested in the concours for the chair of forensic medicine at Strasburg, and gained it over a learned and able competitor. From that time to his death, in February 4, 1835, his application to science and scientific pursuits was unwearied and constant. Amongst his unpublished works are, "Traité des Maladies Nerveuses," 2 vols.; "Philosophie Sociale, ou du Principe de Vie de l'Homme en Société," 4 vols.—*Zeitschrift für Allgemeine Psychiatrie,* vol. iv. part 3.

The Jubilee of Jacobi's Doctorate.—Our German friends have a pleasant custom of celebrating the fiftieth anniversary of some epoch in the life of their acquaintance by a festival or jubilee. This custom is more particularly adopted with reference to the fiftieth anniversary of the doctorate. We subjoin an account of Jacobi's jubilee, and cannot omit the opportunity of expressing our conviction, that the public services of some of our more distinguished professional men might be acknowledged in the same generous manner, with great public advantage. The honour thus paid to venerable worth and genius, would stimulate the young and enterprising to a career of usefulness, and encourage the man of middle-age to active effort, instead of leaving him to struggle on, despairing of reward on this side of the grave, and scarcely hoping that an acknowledgment of his labours will be accorded, when praise and blame fall alike on the dull cold ear of death.—On the 21st of March last, (1846,) the day was celebrated as a festival at Siegburg, on the Rhine, as that on which, fifty years ago, the senior physician and director of the institution, *Ober Medicinal-rath,* Maximilian Jacobi, donned the doctor's bonnet at the then existing University of Erfürt. Although the immediate friends and admirers of the veteran of psychiatry received only a short notice that so favourable an opportunity was to be afforded of testifying their high estimation of him, a great number of friends and comrades were invited from a distance to take part in the festival. And so it happened that it was not local only, but had a general importance. Not only did the Siegburg institution present its congratulations on this day to its first promoter and hoary-headed director—not only did the attendants, patients, friends, and patrons of the hospital bring to the true-hearted physician, and in his field of duty the faithful officer, the assurance of their esteem and attachment; but also the German—nay, the European psychiatry shared in the festival. A multitude of voices, sounding near and from afar, paid to their industrious and meritorious fellow-labourer in the field of psychal medicine the tribute of grateful acknowledgment and honour. Amongst those present at this festival in the interests of science, we may mention the Gehein-rath Wutzer, who, as Dean of the Faculty of Medicine at Bonn, presented the renewed diploma of a doctor of medicine and chirurgery; Professor Argelander, Dean of the Philosophical Faculty at Bonn, who brought the honorary diploma of a doctor of philosophy; the Lower Rhenish Society of Natural and Medical Science at Bonn; the Psycho-Medical

Society of Erlangen, which sent a congratulatory letter; the Medical Society of Hamburg, which forwarded the diploma of honorary associate; and the Medical Faculty of Jena, which presented the diploma of doctor. Further, we may add, Sir Alexander Morison named the appointment of the subject of the festival as a corresponding member of the Society for Improving the Condition of the Insane. A recognition on the part of the government, by the conferring of the Order of a Knight of the Red Eagle of the third class, added to the pleasure of the jubilee.—We find the following particulars respecting the festival in the Rhenish newspapers:—On the evening preceding, Jacobi was surprised by the performance of a serenade, and prepared for the day of honour, of which he himself had no thought. With the early dawn, the procession of officers of the institution was in motion to the house of the jubilist, (jubilars,) to congratulate him; and in it were also the former physicians, and the chaplains and stewards, followed by the subalterns, and a crowd of patients from the hospital. After the ecclesiastics of the two confessions had greeted him, there followed a festive deputation of the civil and military authorities of the city and province, of the Royal Medical College, the general commanding the 8th corps d'armée, the governments of Coblentz, Trêves, Aix-la-Chapelle, and Düsseldorf—Herr V. Laumer, the chief president of the royal government of Cologne, being at their head. Later in the day, the guests sat down to a dinner in one of the saloons of the city of Siegburg, which was enlivened by numerous toasts and festive songs. We must particularly notice the observations made by Dr. M——, formerly an assistant physician at the institution, who proposed to establish a fund, to be called the "Jacobi foundation," out of which a present should be made yearly to the deserving male and female attendants of the establishment, proportionate to their services. The proposition was so much approved, that the sum of ninety-five thalers was subscribed at once, which will doubtless be increased by the contributions of the friends of the establishment residing in the provinces, and by the profits of a work to be published, entitled, "The Jacobi Festival at Siegburg." The festival was concluded by a musical and theatrical performance at the expense of the patients, and by an illumination got up by the patrons. "It was a splendid festival," so concludes the account of it in the "*Rhenish Observer;*" and we repeat the words, because they express the sentiments of all those resident near the place of Jacobi's labours; we repeat them, because they clearly express our own feelings. "It was a fête of acknowledgment for long, unpretending, unwearied, and enlightened activity; an activity, the deep importance of which will only be made fully manifest after a lengthened period, and truly in the hearts of those only who can understand such benevolence of feeling, because they have it themselves. May men never be wanting to the Rhenish province, to Germany, to the whole human race, who, 'gentle and generous,' devote their lives to the service of suffering humanity. Their day of honour will not fail, even although they may not strive for it."—*Zeitschrift für Psychiatrie*, vol. iv. part 2.

